# Supported decision‐making interventions in mental healthcare: A systematic review of current evidence and implementation barriers

**DOI:** 10.1111/hex.14001

**Published:** 2024-03-03

**Authors:** Cathy J. Francis, Amanda Johnson, Rhonda L. Wilson

**Affiliations:** ^1^ University of Newcastle Newcastle New South Wales Australia; ^2^ Head of School, Dean of Nursing and Midwifery University of Newcastle Newcastle New South Wales Australia; ^3^ Massey University Palmerston North New Zealand

**Keywords:** mental health, person‐centred care, shared decision‐making, supported decision‐making, UNCRPD

## Abstract

**Background:**

There is a growing momentum around the world to foster greater opportunities for the involvement of mental health service users in their care and treatment planning. *In‐principle* support for this aim is widespread across mental healthcare professionals. Yet, progress in mental health services towards this objective has lagged *in practice*.

**Objectives:**

We conducted a systematic review of quantitative, qualitative and mixed‐method research on interventions to improve opportunities for the involvement of mental healthcare service users in treatment planning, to understand the current research evidence and the barriers to implementation.

**Methods:**

Seven databases were searched and 5137 articles were screened. Articles were included if they reported on an intervention for adult service users, were published between 2008 and October 2023 and were in English. Evidence in the 140 included articles was synthesised according to the JBI guidance on Mixed Methods Systematic Reviews.

**Results:**

Research in this field remains exploratory in nature, with a wide range of interventions investigated to date but little experimental replication. Overarching barriers to shared and supported decision‐making in mental health treatment planning were (1) Organisational (resource limitations, culture barriers, risk management priorities and structure); (2) Process (lack of knowledge, time constraints, health‐related concerns, problems completing and using plans); and (3) Relationship barriers (fear and distrust for both service users and clinicians).

**Conclusions:**

On the basis of the barriers identified, recommendations are made to enable the implementation of new policies and programs, the designing of new tools and for clinicians seeking to practice shared and supported decision‐making in the healthcare they offer.

**Patient or Public Contribution:**

This systematic review has been guided at all stages by a researcher with experience of mental health service use, who does not wish to be identified at this point in time. The findings may inform organisations, researchers and practitioners on implementing supported decision‐making, for the greater involvement of people with mental ill health in their healthcare.

## 1 INTRODUCTION

Over the last several decades, there has been a growing momentum around the world to develop policies and in some countries, legislation, that mandates collaboration with and greater opportunities for involvement of mental health service users in their care and treatment planning. The UN Convention on the Rights of Persons with Disabilities (CRPD),^1^ which came into force in 2008, has further driven efforts to facilitate people with mental ill health in making decisions concerning their treatment.^2^ In addition, there seems to be general *in‐principle* recognition by mental health practitioners of the importance of shared and supported decision‐making in the care that they provide.^3^, ^4^, ^5^ Yet, evidence demonstrates that progress in mental health services towards this objective, *in practice*, has lagged.^3^, ^6^, ^7^, ^8^, ^9^, ^10^, ^11^, ^12^, ^13^, ^14^, ^15^, ^16^, ^17^

Both shared decision‐making and, more recently, supported decision‐making, are models that can be applied in mental health treatment planning to facilitate opportunities for the greater involvement of service users in that experience.^18^, ^19^ Both sit along a spectrum of approaches to medical decision‐making, between the still prevalent paternalistic model and the autonomous decision model.^20^ In shared decision‐making, both the clinician and the service user are involved in the decision‐making process and both agree on the final treatment to implement, through processes that facilitate the exchange of information and equality in decision‐making.^21^ Supported decision‐making, further along the spectrum towards autonomy, is where a person is ‘provided with necessary supports and accommodation to make and communicate decisions according to his or her wishes … in relation to health care (including mental healthcare) this would mean communicating information about healthcare decisions in appropriate ways, in providing a variety of options, and in understanding and respecting a person's choice’.^22^

Early systematic reviews^4^, ^23^ and more recent meta‐analyses^24^, ^25^ have indicated promising results for the greater involvement of people with mental illness in one type of treatment planning, that is facilitated crisis (or advance) planning. These reviews were based on the few randomised controlled trials (RCTs) conducted to date. Benefits of this type of planning include significantly reduced risk of compulsory psychiatric admission when compared with other intervention types, improved self‐determination and empowerment for service users and benefits for the therapeutic relationship. However, they also flag some potential barriers, particularly in relation to the role of mental health professionals, in the implementation of shared and supported decision‐making in mental health treatment planning.^4^, ^25^

In this broad systematic, integrative review research in the field was explored, beyond crisis planning and RCTs. It aimed to identify quantitative, qualitative and mixed‐method research on interventions to improve opportunities, through facilitation (or support) for the involvement of mental healthcare service users in treatment planning. The following questions informed the review: (i) what research has been undertaken in this field since 2008 when the UN CRPDs came into force, (ii) what are the barriers to the implementation of these types of interventions, (iii) what is the evidence around the associated outcomes for people with mental illness and (iv) how do service users and clinicians experience it? While the systematic review is one that integrates evidence to respond to all four questions as a coherent whole, the overall results are outlined across two articles. In this article, we focus on the findings to the first two questions.

## 2 METHODS

The PRISMA 2020 Checklist^26^ was used to guide the review and the review protocol was registered with PROSPERO (CRD42022340117). Search terms were developed based on significant known key texts, on preliminary pilot searches, and were discussed and agreed among the authors. A search string was designed accordingly, using free‐text keywords and subject headings (or MESH terms). Search terms are shown in Table 1 and the search strings for each database were developed, tailored to each database, as follows: (BLOCK 1) AND (BLOCK 2) AND (BLOCK 3) with limitations as indicated in BLOCK 4 and BLOCK 5.

**Table 1:** Search terms.

BLOCK 1	BLOCK 2	BLOCK 3	BLOCK 4	BLOCK 5
(FT) ‘mental health’	(FT) joint	(FT) ‘treatment plan*’	NOT dementia	Date limited 2008—current
(FT)‘mental illness’	(FT) collaborat*	(FT) ‘care plan*’		
(FT) ‘mental disorder’	(FT) personal*	(FT) ‘safety plan*’		
(FT) psychiatr*	(FT) support*	(FT) ‘crisis plan*’		
(SH) Mental health^1^, ^2^, ^3^, ^4^, ^5^	(FT) facilitat*	(FT) ‘advance directive*’		
(SH) Mental disorders^1^, ^2^, ^3^, ^4^, ^5^		(FT) ‘advance statement*’		
(SH) Mentally ill persons^3^, ^4^, ^5^		(FT) ‘advance agreement*’		
(SH) Psychiatric patients^1^, ^2^		(FT) ‘advance plan*’		
		(FT) ‘advance decision*’		
		(FT) ‘psychiatric plan*’		
		(FT) ‘advance preferences instrument’		
		(SH) treatment planning^2^		
		(SH) nursing care plans^1^		
		(SH) patient care plans^1^		
		(SH) patient care planning^3^, ^4^, ^5^		
		(SH) advance directives^1^, ^2^, ^3^, ^4^, ^5^		
		(SH) advance care planning^1^, ^3^, ^4^, ^5^		

^1^ CINAHL.

^2^ PsychInfo.

^3^ Medline.

^4^ PubMed.

^5^ Cochrane.

Articles were included in the review if the research outlined met all of the following criteria: (a) a mental health treatment planning intervention that facilitated service user involvement in decision‐making, (b) quantitative, qualitative or mixed methods, (c) adult participants, (d) any mental illness/disorder (including substance use disorders), (e) reporting on efficacy and effectiveness of outcomes, experiences or barriers, (f) reporting in English and (g) publication during or after 2008.

Facilitated decision‐making was considered to include processes or practices where assistance by a specified person or digital programme was available to assist service user involvement in their treatment planning. Both shared and supported decision‐making interventions were included. Treatment planning was taken to include any planning related to a person's treatment such as treatment, care, crisis, safety and service planning or a component of such a plan, for example, medication planning.

Articles were excluded from the review if they did not meet all of the inclusion criteria or if the research outlined (a) treatment planning that was a small part of a larger, multifaceted, complex intervention where the impact of the treatment planning could not be isolated, (b) an intervention for people with dementia or intellectual disability and (c) a focus on family or carer experiences.

Databases were searched on 16–17 June 2021 and included CINAHL, Cochrane, Google Scholar, Medline, PsychInfo, PubMed and Scopus. Google Scholar was a free‐text keyword search only, and due to the character limit had to be divided into three separate searches: (BLOCK 1) AND (BLOCK 2) AND (PART OF BLOCK 3 [i], [ii] and [iii]), excluding ‘dementia’ and specifying the required date range. The top 100 results from each Google Scholar search were captured. An updated search of the same databases with the same search string was undertaken on 9 October 2023.

Search results were extracted to a Covidence library, to manage articles. The screening was conducted by one team member (C. F.), with discussions held with other team members (R. W. and A. J.) when necessary to determine articles for inclusion or exclusion. Although review‐type publications did not meet inclusion criteria, references in these publications were also screened to extend coverage, and any relevant references were assessed against the criteria.

To answer the question (i) above, data were extracted from the articles included in the review into a standardised data extraction pro forma in Excel. Design of the pro forma and data extraction was undertaken by one team member (C. F.) and reviewed by two senior team members (R. W. and A. J.). Data collected included location of research and date of publication, methodology used, research settings, characteristics of the sample populations and characteristics of the interventions made. These data were collated, summarised and are presented descriptively.

Due to the heterogeneity of research methodologies, the large number of articles for review and as no research was to be excluded from the review on the basis of quality, a formal quality assessment (e.g., using CASP or JBI tools) of articles was not undertaken. However, in the standardised data extraction pro forma, information relevant to quality of the research was collected for general observation. Data were collected on ethics approvals, whether service user groups were included in research planning, if research questions/objectives were stated, on sample sizes and whether recruitment data were provided, if data analysis methods were specified and if study limitations were outlined. These data were also collated, summarised and are presented descriptively.

To address literature review questions (ii)–(iv), the JBI Manual for Evidence Synthesis guidance on Mixed Methods Systematic Reviews^27^ was followed. In line with this guidance, the articles included in this review were analysed using a ‘convergent segregated approach’ because the aim of this review was to integrate the findings to all three questions (ii)–(iv). According to the convergent segregated approach: the quantitative and qualitative data arising from the articles included in the review are synthesised independently in the first instance, then subsequently integrated. Qualitative data are pooled through meta‐aggregation, to develop categories that aggregate like findings and that can then be synthesised to form ‘an overarching description of a group of categorised findings’. Quantitative data are synthesised, in this case by a narrative summary (as meta‐analysis is not possible). The quantitative and qualitative evidence is then integrated to produce an overall ‘configured analysis’, where the complementarity (or not) of the findings are considered by ‘using one type of evidence to explore, contextualise or explain the findings of the other type of evidence’ to ultimately organise results into a coherent whole.^27^

The findings of the review are presented across two papers, of which this is the first.

## 3 RESULTS

**Figure 1:**
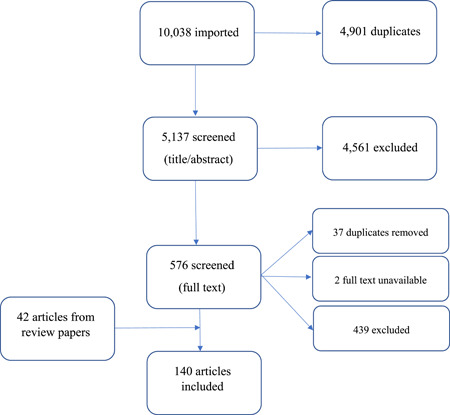
PRISMA flow chart.

A total of 140 articles were included in the review. Database searches identified a total of 10,038 articles, from which 4901 duplicates were initially removed. A further 4561 were subsequently excluded following title/abstract screening, with an additional 478 removed following full‐text screening. While review‐type publications were not included, the reference lists were screened and, as a result, an additional 42 articles were included in this review. The PRISMA flow chart is shown in Figure 1.

### 3.1 Current evidence

Question (i) asked what research has been undertaken since 2008 on facilitating the involvement of service users in mental healthcare and treatment planning. The research undertaken has mostly used quantitative design (75 articles), with qualitative (34 articles) and mixed‐method designs (30 articles) also used (Supporting Information S1: Table 1). Although 40 studies describe interventions that have progressed to testing in randomised controlled trials, research has been primarily exploratory in nature, with a wide range of intervention types, tools and parameters investigated. Among the few more common intervention types are various forms of Care Plans (24 articles), Joint Crisis Plans (17 articles) and Psychiatric Advance Directives with facilitation (17 articles). Research has slightly favoured non‐crisis over crisis treatment planning (53% vs. 42%, respectively) (Supporting Information S1: Table 1). Where reported, length of interventions could be 1, 2, 3 or ≥4 sessions (up to 12 sessions), taking from as little as 15 min up to approximately 1 year to complete a plan. Most of the research has focused on various clinicians as facilitators, although other facilitators studied have included peer support workers, patient advocates, lawyers and digital tools. Most articles focused on people with serious or severe mental illness, and mostly in community‐based settings (Supporting Information S1: Table 1). A growing number of relevant digital tools are covered in the literature (26 articles), and most digital interventions were provided along with additional human support. The majority of research has been on facilitating people with mental illness to play a greater role in decision‐making but where the final decision is shared  (e.g., shared decision‐making). Supported decision‐making in line with the definition provided in the introduction above is relatively little studied to date (Supporting Information S1: Table 1).

### 3.2 Research quality

Research quality can be described as mixed. Most articles specified the research aims (or questions, hypotheses, objectives) and data analysis methods used. However, over 15% did not address ethics approval requirements, almost 60% provided only partial or no recruitment data, most articles used convenience sampling (which increases the potential for bias and limits generalisability) and the majority involved relatively small sample sizes (less than 100 participants). Just 10% of articles involved service users in research planning (as distinct from being involved in designing the intervention or being research participants) and only 20% published templates (in full or in part) for the plans or tools they used in their interventions (making independent replication difficult).

### 3.3 Barriers

The qualitative and quantitative data presented across the 140 included articles were analysed independently and then integrated to form the following findings on the barriers that exist to shared and supported decision‐making in mental health treatment planning. The quantitative evidence generally supported or was consistent with the qualitative evidence reported. Based on the analyses conducted, three overarching types of barriers are identified as follows:

1. Organisational barriers,
2. Process barriers and
3. Relationship barriers.

1. *Organisational barriers*
  The review of the research indicates that *the organisation matters*. Organisational resource limitations, culture barriers, risk management priorities and structure can all act as barriers to facilitated decision‐making in mental health treatment planning. For example, limited organisational financial resources and investment in services and staffing affected staff availability, high staff turnover and workloads: ‘we're short of money and … we're short of people’^28^ were issues reported to affect clinicians' ability to undertake shared and supported decision‐making when treatment planning with service users.^29^, ^30^, ^31^, ^32^, ^33^, ^34^, ^35^, ^36^, ^37^, ^38^ These barriers were further exacerbated by the Covid pandemic.^39^ Also, training costs can be substantial^35^ and, when insufficiently allocated, could mean that ‘the scene was not set for clinicians’,^21^ when they did not receive sufficient information, training or support to understand and adopt such new interventions.^36^, ^40^ Organisational culture and particularly a lack of readiness to change were also barriers—when organisations and leadership avoided a sustained investment in change, did not involve frontline staff in implementation processes or support the necessary changes to address staff work priorities and availability, IT infrastructure, physical infrastructure (i.e., the need for private spaces), clinical workflows and access to guidelines.^29^, ^34^, ^36^, ^38^, ^39^, ^40^, ^41^, ^42^, ^43^, ^44^, ^45^, ^46^ In addition, balancing organisational risk management priorities with increased service user involvement in decision‐making could be challenging and a concern for practitioners.^45^, ^47^ Organisational structure was another barrier to the broad uptake of a new intervention, for example, where a decentralised system meant that consistent cross‐site policies and procedures were not possible across a jurisdiction.^44^ Service (and associated staff) reconfigurations^37^ as well as communicating and collaborating between agencies^31^, ^36^ and within site among multidisciplinary teams can also be problematic.^43^, ^48^ For one culturally specific intervention and the workers implementing it, compatibility with conventional organisational medical models and teams was also reported as a barrier.^49^
2. *Process barriers*

Key barriers that affected the process of facilitated decision‐making in treatment planning included a lack of knowledge; a lack of time; concerns related to the health status of the service user; problems with completing the plan; and problems using the plan. For example:

(i) *Lack of knowledge*
  Without information or training, both service users and clinicians can lack knowledge of^42^, ^46^, ^50^ or experience with^32^ shared or supported decision‐making in treatment planning. A lack of knowledge can negatively impact both clinicians and service users in their understanding and valuing of the purpose or function of such planning,^33^, ^50^, ^51^ how to communicate within and about this type of care and planning,^33^, ^36^, ^52^ how the plans are completed or documented^36^, ^51^, ^53^, ^54^ and how the planning process is facilitated and experienced.^36^, ^46^, ^50^ Even with training, knowledge can wane over time^55^ and attitudes may not shift—following specific training on the power shifts necessary to achieve shared decision‐making, many clinicians (>40%) still believed that the decision‐making power balance should favour the clinician.^54^ When digital tools were used to facilitate the process, additional barriers were experienced by some clinicians and service users, in not knowing how to use the technology generally as well how to use it in the therapeutic relationship.^28^, ^46^, ^52^, ^56^
(ii) *Time constraints*
  Both actual and perceived time constraints were an often‐reported barrier to the process of facilitated decision‐making in treatment planning.^33^, ^34^, ^36^, ^37^, ^43^, ^46^, ^47^, ^52^, ^54^, ^57^, ^58^, ^59^, ^60^, ^61^ For some interventions, the process was reported as time consuming, mostly by clinicians^30^, ^48^, ^51^, ^62^, ^63^ but also by some service users.^32^ Sufficient time was not only needed for the treatment planning session/s with service users but also for clinicians beforehand to learn a new tool and approach, as well as for collaboration with colleagues when needed.^33^, ^36^, ^37^, ^45^, ^63^ Service users were acutely aware of time constraints on practitioners and it affected how some experienced the process.^59^
  like, just getting the laundry done is a hassle, so for me to take someone, sort of, offline and concentrate on me and talk about – I need someone who's got the time for that … otherwise I'm just going to clam up … they've got too much to do …. you don't have time for sitting and listening to me.^50^
(iii) *Health*
  One of the larger studies found that only a very small proportion of people felt that they were too unwell to complete a facilitated plan (9/285 = 3%)^64^ and another similarly reported that of 128 observations, most service users were found (by clinicians) to be capable of, interested in and able to understand the shared, medical decision‐making process.^52^ However, more broadly across qualitative studies, both service users and clinicians reported that at times, service users ill health could be a barrier to being involved in planning.
  Clinicians described some service users as having difficulty following the information, that those in acute crisis tended to be more difficult to engage with, that some had cognitive problems and were easily overwhelmed, did not recognise how ill they were or were just too unwell.^28^, ^36^, ^48^, ^52^, ^58^, ^63^, ^65^, ^66^ In this context, clinician concerns were framed as the patient and their health status being the ‘most significant barrier’ to their involvement in treatment planning,^48^ rather than the barrier being the level of support that could be provided by the clinician. While on the one hand clinicians commonly viewed service users as the main barrier to shared decision‐making in treatment planning due to their health, at the same time, they held the expectation that service users should take the initiative and responsibility for driving the process.^29^, ^30^, ^32^, ^48^ In one study, clinicians also reported that service users could sometimes have other (physical) health problems that interfered with shared decision‐making.^52^
  From the other perspective, some service users felt that sometimes, they could not trust in their ability to engage in dialogue with clinicians, doubted their own perspective, had to regain trust in their own thinking, felt that they may not recognise how serious their illness was, had difficulty following the discussion, completing forms or using technology (i.e., symptom‐related fears when digital tools were involved) or just were not feeling well enough at that point in time.^21^, ^29^, ^37^, ^42^, ^45^, ^46^, ^66^, ^67^, ^68^ It is noted that none of these circumstances preclude a person being more involved in their treatment planning, rather indicating when additional support may be required.
  I have to ask myself whilst [talking to clinicians about treatment decisions] are any of these ideas delusional, are they psychotic? Actually to be honest, once people start talking to you about delusions and psychosis and a lack of insight, you don't half begin to doubt yourself. So yeah, I think I'm probably okay, but I'm having to… regain my trust I suppose in my own thinking. (Male, Service User, Interview)^21^
  Being too well could also be a barrier to undertaking facilitated treatment planning. Service users might feel that they had not experienced a ‘genuine crisis’, were sufficiently improved, may not want to recall or were embarrassed about previous periods of illness, increasing their reluctance to take part.^32^, ^42^, ^46^, ^61^, ^66^, ^67^, ^69^
(iv) *Completing a plan*
  In the context that most service users wanted to be involved in planning for their care, much more so than handing over decision‐making to another person^70^—some may feel hesitant, lacking confidence to begin,^46^, ^71^, ^72^ and can find aspects of completing a plan to be difficult. Difficulties can be experienced with long, repetitive, detailed or legal documents (i.e., some psychiatric advance directives), challenging language or questions, not understanding the document or knowing what to write, also with problem‐solving, deciding on actual preferences and completing the plans.^32^, ^38^, ^42^, ^44^, ^46^, ^52^, ^73^, ^74^, ^75^ Some healthcare providers can also find some plans to be too long^62^ or the process too elaborate.^76^
  Although in one study most service users preferred some guidance in the form of a template, it was also noted that a treatment planning document that looked too bureaucratic and rigid was off‐putting.^42^
  Yeah, that [referring to one of the three AS forms shown in the focus group] just looks like a normal form that the government gets us to fill out every couple of weeks when we're—couple of months when we're being check [sic] up upon […] whereas people aren't going to, you know? They're going to go, ‘Oh, hang on, I'm still not going to trust this again’, and throw it out. Why bother?^42^

Clinicians too found plan templates without flexibility challenging to complete.^77^ It may also be difficult to find a balance between information that service users are able and comfortable providing and which they want to relay, versus what healthcare professionals find useful, particularly in a crisis.^42^, ^78^, ^79^

The way a plan was introduced and provided to service users was also important—simply providing written information for the service user to take home and read was not a successful strategy and trying to complete a plan by providing support over the phone could also be problematic.^42^ Cultural and language differences could also be a barrier, from the clinician's perspective, that sometimes hindered the shared decision‐making experience.^33^, ^49^, ^52^

Involving different parties (including colleagues and family members) in the planning process was another barrier that has been reported as being difficult, or at least perceived as difficult.^31^, ^36^, ^65^, ^69^

Additional barriers to plan completion may also be experienced by (a) younger people, in one study those under median age 42 years were less likely to complete a plan compared with their older counterparts^53^; (b) people discharged, relocating away or not receiving care from services where such planning occurred^32^, ^80^; (c) people who were experiencing homelessness and poverty;^42^, ^69^ and (d) although less education was shown to be associated with higher rates of plan completion,^81^ illiteracy was viewed by both service users and providers as a potentially significant barrier to planning involvement and completion, and may influence whether clinicians offer such planning opportunities.^28^, ^42^, ^45^, ^56^

(v) *Use of the plan*

Service users were concerned about how such plans would be used, when the purpose of the planning intervention was not explained to them and they did not trust mental health professionals to use it in a way that would benefit them.^50^, ^82^ In addition, some clinicians and service users reported perceptions that the plans would lack usefulness.^40^, ^50^, ^69^, ^83^

Clinicians also reported concern that if they helped to prepare a plan with a service user, they could not ensure that future treating professionals would use and honour the plan and that this would undermine the process.^21^ Service users too worried whether the plan would be respected and used by their healthcare team.

… if it's not followed through by the doctors… … Yeah, it's going to create a bigger… So, in a sense that would make it even worse for someone trusting the process [… Trust] It's a massive part [of the process].^42^

It seems that these concerns were not unfounded. When investigated, it was relatively common for people's plans not to be reviewed after completion^42^, ^50^, ^78^, ^80^, ^84^, ^85^ or followed by treating clinicians,^36^, ^50^, ^61^, ^81^, ^84^, ^86^, ^87^ even more so if a person was involuntarily admitted into care.^81^ The research indicates that preferred care as outlined in a plan was not followed by clinicians: without reason, because the service user was deemed to have disengaged or not followed the plan, because it was deemed not to be in the service users' best interests, because nominated support people/decision‐makers were unavailable on the day, because staff did not look for such a plan or if they did come across one, they did not understand its purpose and because the treating team could not agree on how to implement the plan. The implications of decisions made by the healthcare team not to follow plans were impactful on service users. For example, people were involuntarily admitted when their plan specified other reasonable alternatives, or were given injectable depot medication when their plan refused injections.^84^

Another significant barrier to these plans being used was a lack of accessibility, where and by whom they were needed.^21^, ^40^, ^42^, ^44^, ^61^, ^84^, ^86^, ^88^ Relatedly, plans were generally not well integrated with the electronic medical records for ease of access.^42^, ^44^, ^61^ Also, in some studies, it was common for clinicians not to provide service users with a copy of their plan.^42^, ^89^ This is problematic, as some service users reported having trouble recalling details of their plan;^88^, ^90^, ^91^ they therefore needed to be able to access and share it with people of their choosing. At the same time, service users reported difficulty keeping track of a piece of paper^83^ and had concerns about privacy and confidentiality.^46^, ^83^, ^86^

For plans that were accessible, those that were incomplete or inadequate, lacked information on medication and lacked contact details for key people were clinician‐rated as being less useful in follow‐up reviews or during a crisis.^31^, ^36^ Conversely, though, clinicians thought that if the plans were too long, they would also not be useful, particularly in an emergency.^42^

3. *Relationship barriers*

*Fear and distrust* were prominent, impacting on relationships, and as a result were a significant barrier to shared and supported decision‐making in treatment planning. For example:

(i) *Service users*
  Some people with mental illness did not want to engage in treatment planning, or were opposed to taking part.^37^, ^54^, ^58^, ^64^, ^92^, ^93^ For those people who did not participate, and even for the service users who did, *distrust*,^21^, ^30^, ^32^, ^42^, ^47^, ^93^, ^94^ *fear* ^47^, ^50^, ^89^ and *disempowerment* ^21^, ^29^, ^32^, ^42^, ^47^, ^84^, ^95^ of and by clinicians and the system were reported—based on people's previous negative experiences^38^ of being disrespected (see below), coerced^32^, ^42^, ^47^, ^53^, ^88^ and not having their needs met^29^, ^70^, ^73^ in treatment.
(ii) *Mental health professionals*

There were widespread reports of service users having negative interactions with their clinicians, both in the past and during facilitated treatment planning, which presented a substantial barrier to undertaking this kind of intervention. For example, clinicians were reported as being disrespectful of service users by being inconsistent (saying or promising one thing and doing something else), condescending, patronising and judgemental, by not listening, not taking the time to explain matters and not respecting service users' wishes.^29^, ^42^, ^47^, ^50^, ^51^, ^64^, ^71^, ^84^, ^86^, ^96^, ^97^

Furthermore, many clinicians were resistant to changing their practices to accommodate shared or supported decision‐making in treatment planning. For example, in one study, no psychiatrists went to service‐wide training attended by 350 other health professionals.^3^, ^41^ Also, in one large randomised‐controlled trial, many clinicians did not follow the intervention protocol,^97^ many did not engage in the process^21^ and for those who were exposed to the intervention, it did not result in subsequent changes to planning documents.^98^

… But I remember, on the day that [the facilitator] came [the psychiatrist] was on the [computer], he was so rude […] and he was on his [computer] most of the time when [the facilitator] was talking. He had his back turned.^21^

[The JCP Facilitator] had made an appointment and turned up and [the psychiatrist] refused to let her in to my meeting, he said ‘not on my time’ he said.^47^

Clinicians not following or engaging in the intervention protocol was similarly reported in other studies.^29^, ^33^, ^37^, ^46^, ^51^, ^52^, ^59^, ^74^, ^99^, ^100^ More experienced clinicians were found to be associated with lower completion rates of crisis plans with service users.^81^ In addition, several studies reported that substantial effort or support was required for clinicians to change their approaches and routine practices.^57^, ^101^

One obstacle may be that many clinicians believe that they are already doing shared decision‐making in treatment planning^21^, ^30^, ^32^, ^33^, ^34^, ^46^, ^47^, ^54^, ^97^—although when they describe their practices, it suggests otherwise.^97^ Clinicians also acknowledged that they had other priorities,^31^, ^46^, ^60^ were sceptical about the associated benefits^21^, ^61^, ^76^, ^97^ and found shared decision‐making a challenge, including emotionally.^36^, ^38^, ^40^, ^52^, ^58^, ^102^ For example, in one intervention, service users who were trained in shared decision‐making were perceived by clinicians to be ‘more difficult’.^103^ Also, on the one hand, while clinicians found it difficult that some service users were less motivated to take part, on the other hand, when service users were more involved, clinicians also found it problematic when there was disagreement^36^—including holding concerns that service user decisions might conflict with perceived obligations for beneficence.^21^ Furthermore, clinicians were concerned with the additional effort and engagement required,^33^, ^34^, ^59^ the limited care pathways available,^21^, ^32^, ^52^ that they would not be able to follow through on a person's wishes^47^ and that risk/organisational management requirements specifically undermined the therapeutic relationship.^41^, ^47^ That is, clinicians were fearful too—of outcomes and the process, of how service users, therapeutic relationships and their careers would be impacted. Experiences of imposing involuntary treatments also made some feel uncomfortable about subsequently talking with service users about supported decision‐making.^89^ In addition, there were some clinicians who expressed views that they thought shared decision‐making should not involve clinicians,^47^ that this type of treatment planning is unnecessary or pointless,^40^, ^48^, ^76^ that service users do not want to engage in the process^21^, ^54^ or that they want to engage too much,^52^ they will not accept their diagnosis, are indecisive and cannot handle the process^52^ and that service users already know what to do.^21^

As a result, key elements of facilitated decision‐making did not occur. For example, service users' knowledge was not drawn upon^29^; collaboration did not occur;^36^, ^69^, ^97^ service users were presented with limited options or ‘choices’ that were not really choices'^21^, ^47^ and goal‐setting reflected mental health professionals' aspirations rather than service user needs and expectations.^58^ Simply attempting to undertake shared or supported decision‐making in treatment planning did not guarantee an improved therapeutic alliance^84^, ^92^; if not done well, it could further expose those relationships that were not working.^30^

### 3.4 Comparison of qualitative and quantitative evidence

When comparing the qualitative and quantitative evidence on the barriers to shared and supported decision‐making in mental health treatment planning, two key observations are made. The evidence on organisational‐ and relationship‐type barriers to date has been primarily qualitative in nature and could therefore be strengthened by future quantitative research in these areas. Evidence on process‐type barriers was more balanced between both qualitative and quantitative results. However, both of these observations need to be considered in the context of one of the biggest gaps in research to date, that there is a need to further develop a stronger replicated body of evidence on any one specific tool.

## 4 DISCUSSION

Research to date in this field can be described as primarily exploratory in nature, with a wide range of intervention types and parameters investigated, and little replication. More work is needed to consolidate the findings of that exploratory research, and to now extend investigations into supported decision‐making. However, from the body of knowledge generated by the research included in this review, it is clear that numerous barriers exist to the greater involvement of people with mental ill health in care and treatment planning, that is, to shared and supported decision‐making in mental healthcare.

Three main barriers were identified: Organisational barriers, Process barriers and Relationship barriers, with notable overlap existing between them. Central to and underlying all three of these barriers were the common and intertwined threads of fear and distrust—fear of the unknown, of change, of not meeting expectations, of past negative experiences reoccurring and of potential consequences, as well as distrust in one's self, distrust in others, the system and in the benefits of shared and supported decision‐making in treatment planning.

Distrust has been identified as a key barrier for other new interventions in the mental healthcare field. For example, Tørseth and Ådnanes^104^ found that in the implementation of a new care pathway approach to mental health services in Norway, distrust by clinicians played a considerable role in creating resistance to the adoption of the new approach. Similarly, for service users, distrust has been identified as a key barrier in mental healthcare, where coercion^105^ and the fear of negative consequences as well as lack of fairness, transparency and accountability^89^ impact on a service user's trust in their care provider and the system.

It follows, then, that minimising these barriers and providing the right organisational, process and relationship assistance are key to successful shared and supported decision‐making in mental health treatment planning. With the need to develop trust at the heart of the actions to be taken. For example, in order to take part in shared or supported decision‐making in treatment planning in the first instance, service users say that they must be able to trust the professional who is facilitating the process,^29^, ^42^ while clinicians need to be able to trust that their organisation supports and prioritises this type of intervention, that service users want to be involved, that service users can and do make reasonable requests and decisions and that it is feasible and of benefit. Coulter^106^ highlighted that this type of decision‐making is more than just a process; it is also a relationship, and the foundational need for trust as a key facilitator of shared decision‐making has been identified in numerous other fields including oncology, paediatrics and indigenous health.^107^, ^108^, ^109^

### 4.1 Review limitations

The results of this review need to be considered in the context of its limitations. While some data relevant to quality of the research reviewed were captured and are presented descriptively, a formal quality assessment of the included articles was not undertaken. In addition, only English‐language publications were included, therefore excluding any research described in other languages and potentially under‐representing studies from low‐ and middle‐income countries. The review is also limited to research on adults seeking mental healthcare and therefore does not consider efforts to implement shared and supported decision‐making for children and young people.

## 5 CONCLUSIONS

Three recommendations arise from this review, corresponding with the three types of barriers identified. First, when implementing *new programs or policies* that seek to introduce and embed supported decision‐making into routine care practices, particular attention must be paid to reducing *organisational* barriers. Sufficient staffing levels, time and education are especially important and must be available so that clinical staff are themselves supported by and trust in the organisation, in order to undertake this type of decision‐making with service users. If lacking, innovation will be difficult to translate into widespread implementation, as has similarly been reported for other health initiatives.^110^, ^111^, ^112^ Tools to assist supported decision‐making in routine care will be useful in this setting.

Therefore, we also recommend that during the *design of new tools* for the purpose of supported decision‐making, developers must as a priority seek to reduce the *process* barriers identified in this review. The length of time that it takes to complete a facilitated treatment plan is no doubt a key issue to be addressed and further investigated, noting that interventions taking as little as 15 minutes have already been designed.^113^ In addition, how people understand, view, use and trust any such tool will be critical in its success—involving service users and service providers in co‐design of new tools can help address these factors.^114^, ^115^ New tools must also accommodate people's dynamic health status and the associated flexibility required in the support offered, as well as consider how any care or treatment plan created through the tool will be accessible to those who need it, when and where they need it.

*For clinicians* wanting to incorporate supported decision‐making into their practices, a particular awareness of the *relationship* barriers identified will be necessary, as will attempts to reduce them. A commitment to genuinely reflect on and adjust practices, perceptions and power balances, and to seek ways to help reduce service user fear, distrust and disempowerment, will be critical. Laugharne et al.^116^ found that from service user perspectives, clinician features that help develop trust include a willingness to listen, honesty, reliability, kindness and being positive about the future. While empowerment will surely be facilitated in a respectful relationship in which human rights, as per the CRPD, are central and upheld.

## AUTHOR CONTRIBUTIONS

**Cathy J. Francis**: Conceptualization; investigation; writing—original draft; methodology; writing—review & editing; formal analysis; data curation; project administration; validation. **Rhonda L. Wilson**: Conceptualization; methodology; writing—review & editing; formal analysis; project administration; supervision; validation. **Amanda Johnson**: Methodology; writing—review and editing; supervision; project administration; validation.

## CONFLICT OF INTEREST STATEMENT

The authors declare no conflicts of interest.

## Supporting information

Supporting information.

## Data Availability

The data that support the findings of this study are available from the corresponding author upon reasonable request.
